# Best Practices for The Interdisciplinary Rehabilitation Team: A Review of Mental Health Issues in Mild Stroke Survivors

**DOI:** 10.1155/2018/6187328

**Published:** 2018-06-04

**Authors:** Alexandra L. Terrill, Jaclyn K. Schwartz, Samir R. Belagaje

**Affiliations:** ^1^Department of Occupational & Recreational Therapies, University of Utah, Salt Lake City, USA; ^2^Occupational Therapy Department, Florida International University, Miami, FL, USA; ^3^Departments of Neurology and Rehabilitation Medicine, Emory University School of Medicine, Atlanta, GA, USA

## Abstract

Individuals with mild strokes are generally considered fully functional and do not traditionally receive rehabilitation services. Because patients with mild stroke are assumed to have a good recovery, they may have deficits in other areas, including mental health, that are not addressed. As a result, patients with mild stroke are unable to meet quality of life standards. In addition, healthcare professionals are likely unaware of the potential mental health issues that may arise in mild stroke. To address this gap in knowledge, we review the evidence supporting mental health evaluation and intervention in mild stroke. Specifically, we review comorbid diagnoses including depression, anxiety, fatigue, and sleep disturbances and their potential effects on health and function. Finally, we conclude with general recommendations describing best practice derived from current evidence.

## 1. Introduction

Half of all strokes are mild in nature [1]. Traditionally, the most common way to identify persons with mild stroke are the NIH stroke scale (mild stroke defined as NIHSS < 5) and the modified Rankin (mild stroke defined as mRS < 2). While these tools are of historical significance, they focus on physical ability and do not fully capture the cognitive and emotional issues of stroke survivors. Subsequently, individuals with mild stroke may score as low as 0 on the NIHSS and the mRS while experiencing stroke sequela that negatively impact their ability to return to their complex everyday lives [2, 3]. Despite best research, current clinical practice often represents the historical view of persons with mild stroke, with most patients reporting little to no follow-up services [4]. With advances in the acute treatment of stroke, the number of mild strokes will continue to increase [1]. Healthcare providers must update their practice approaches to rehabilitate the body and minds of persons with mild stroke, such that they can return to healthy productive living.

Mental health is one aspect that negatively impacts persons with mild stroke and their caregivers [5]. Traditionally, these patients are assumed to have a good recovery and subsequently they are not evaluated and treated for mental health concerns. One reason for the oversight of the mental health of mild stroke survivors is that healthcare professionals are often unaware of the potential mental health issues that may arise in mild stroke. In addition, mild strokes and TIAs may serve as indicator of general cerebrovascular disease which over time can lead to mental health and cognitive issues not readily apparent on bedside testing or imaging [6]. The purpose of this paper is to improve best practices by educating rehabilitation clinicians on the mental health issues in mild stroke including depression, anxiety, fatigue, and sleep disturbances. We will conclude with recommendations for evaluation and treatment.

## 2. Mental Health Conditions after Mild Stroke

### 2.1. Poststroke Depression And Anxiety

Poststroke depression (PSD) is a common complication that occurs in approximately one-third of stroke survivors [25]. PSD is characterized by symptoms consistent with depression, including low mood, anhedonia, changes in appetite, concentration, decreased energy, and change in sleep. PSD more frequently occurs in the acute stages of stroke (early-onset) and remits within the first year, but it can also occur during the chronic stages (late-onset). Major risk factors for PSD are severity of stroke and disability after stroke [1]. Despite being characterized by mild, short-lasting symptoms and relatively low functional impairment, prevalence of PSD in mild stroke is comparable to PSD in stroke in general, with estimated rates ranging from 29% to 40% [26–28]. Research suggests that being female, smoking, and mild cognitive impairment are associated with a high risk of acute-onset PSD [27]. Being female, smoking, cognitive impairment, pain, therapy enrollment, low social support and community participation, and stroke recurrence are associated with higher risk of PSD in chronic mild stroke [27–29]. Prestroke depression may [25] or may not be associated with PSD [30].

Poststroke anxiety is another common experience with prevalence estimated at 20%-47% [29, 30]. It may present as generalized anxiety or stroke-specific. For example, survivors often contend with changes in functional abilities and navigating life situations in addition to having experienced a serious, life-threatening medical condition with risk of recurrence, which may contribute to anxiety. Because of the relatively mild consequences of minor stroke, survivors are often considered to have recovered and face expectations they may not be able to live up to as a result of hidden or invisible dysfunction due to emotional difficulties (PSD, anxiety) or fatigue [31]. Poststroke anxiety is significantly associated with prestroke anxiety and PSD as well as less disability [29, 30].

A specific type of poststroke anxiety is posttraumatic stress disorder (PTSD). Prevalence of posttraumatic stress disorder following stroke is estimated at 6-31% of survivors [32–35]. Symptoms of poststroke PTSD are persistent and may include intrusive thoughts or recollections of the event, having nightmares, avoidance or numbing, hyperarousal (feeling “on guard”, easily startled), sleeping difficulties, having increased anxiety, or having anger outbursts. Being female, younger age, an intense peritraumatic reaction (i.e., fear during the stroke), having a history of depression or anxiety, and cognitive appraisals are significant predictors of both the number and severity of PTSD symptoms following stroke [33, 35, 36]. However, these findings are not specific to mild stroke and further research is needed in this area.

### 2.2. Fatigue

Fatigue is another important adverse consequence of stroke whose importance is being more recognized [37]. Poststroke fatigue manifests itself in two types; one type is a physical fatigue as patients are not able to perform at physical lengths and intensities. There is also a mental fatigue component which affects concentration, ability to multitask, and dealing with stressors. It is the latter which comprises mental health issues. In a qualitative study of a focus group of 19 mild stroke survivors, Flinn and Stube (2010) reported that survivors reported feeling unprepared to deal with poststroke fatigue [38]. The study participants reported adverse effect in daily activities including social participation, return to work, driving, reading, and sleeping. In quantitative studies of both young and old mild stroke survivors, fatigue was reported as the most common complaint [39, 40].

The literature states that the prevalence of fatigue in minor stroke is between 23 and 40% without significant statistical differences in the demographics of the cohort with fatigue and those without it [41]. The numbers may be overstated as the studies incorporated transient ischemic attacks (TIAs) as well; furthermore, the difference in numbers amongst the studies may be due to the measurement tool used to measure fatigue. There is a statistically significantly higher prevalence in minor stroke compared to TIAs which suggests that there is a biological effect of the stroke [37]. However, as all these studies show, fatigue is seen in TIA patients which also suggests that there are other factors in the development such as medication side effect and lifestyle modifications. In addition, many patients diagnosed with TIAs actually demonstrate evidence of brain damage as seen on MRI and have general vascular risk factors which can contribute.

The literature also reports that once fatigue develops, it does not resolve spontaneously. Radman et al. studied the prevalence of fatigue in a longitudinal study and measured fatigue using the Fatigue Inventory Assessment in 95 stroke survivors with minor stroke (nondisabling, NIHSS< 6). They found that the prevalence of fatigue at 6 months after stroke was 30% and at 1 year after stroke was 35% [42]. At 1 year, 11% of the cases were new reports which suggests that the majority of patients who were fatigued at 6 months did not improve.

### 2.3. Sleep Disturbances

Sleep disturbance after stroke is another aspect of adverse mental health. Poor sleep has been attributed to poststroke mental health issues such as fatigue, poststroke depression, and cognitive impairment [43–45]. The sleep issues are diverse and can represent trouble with insomnia, sleep apnea, sleep efficiency, and/or excess sleep issues [43]. The majority of the literature to date has all stroke severity classes or focused on those in an inpatient rehabilitation setting, a setting in which mild stroke survivors are underrepresented after the first days in the acute setting.

Yet mild stroke survivors suffer from poststroke sleep disturbances and the sparse literature focused exclusively on this population provides evidence. In a group of 80 stroke survivors less than 2 weeks out from stroke onset with mild stroke defined as modified Rankin Score ≤ 3, sleep quality was measured using three different scales [46]. Depending on the scale used, anywhere from 49 to 71% reported sleep disturbance issues. Another interesting aspect of this study was that those who reported poor sleep were also found to have lower motor functional scores measured by a battery of tests compared to those who had better sleep. This finding underscores the importance that sleep has on overall poststroke outcomes, even in mild stroke. Similar to poststroke fatigue, sleep issues did not improve over the course of a year in mild stroke survivors [47].

## 3. Pathophysiology

The etiology and development of the mental health issues associated with mild stroke are not clear and remain a source of debate. Mild strokes are often associated with small sized strokes and/or locations in nonessential strokes. Furthermore, unlike hemiparesis or visual deficits, the poststroke mental health deficits do not localize well to definite brain locations [42, 48].

These findings seem to suggest that the mental health issues associated with mild stroke can be largely attributed to psychosocial reactions that may be interacting with biological mechanisms. There is also some evidence for this finding. In an interview of 18 mild stroke survivors 1 year after stroke, there were issues to adjustment with stroke [39]. In particular, the interviewees struggled to “manage an everyday life of uncertainty” as they navigated worries about stroke recurrence, recovery from their symptoms, the uncertainty about employment, and relationship concerns. In another study, patients had a dramatic change in self-perception regarding their health and ability to participate in social activities [49]. A similar negative self-perception in regard to ability to work, fatigue, and dependence was also demonstrated in a survey of young mild stroke survivors and associated with overall decrease in perception of global health [40].

In addition, many of the poststroke mental health issues are related and intertwined. For example, poststroke sleep disturbances will cause fatigue and depression can manifest as sleep disturbances and low energy or fatigability. Moreover, mild stroke survivors may become more dependent on caregivers, which leads to changes in relationships, changes in social roles, and in turn, an altered sense of self and potentially greater risk for depression and lower quality of life for both survivor and caregiving partners. [50, 51].

On the other hand, there still may be a biological basis for these findings. Strokes often result in an inflammatory responses and lead to a rise in inflammatory markers. Research has shown an increase in inflammatory cytokines such as IL-6 in mild strokes [52]. These inflammatory markers are seen in other systemic conditions such as autoimmune diseases. Therefore, it should not be surprising that many people with these conditions experience similar symptoms such as fatigue, sleep disturbances, and emotional disorders. Inflammatory and endocrine responses as well as autonomic reactions can also be induced as stress reactions. There is also evidence that inflammatory mechanisms contribute to depression [53, 54]. A full discussion of the interaction between inflammation and mental health disorders is beyond the scope of this review but can be found in other sources [55, 56].

Another biological basis to consider is that mild strokes can lead to disconnection of widely distributed networks in the brain. Clinical correlates of these poststroke disconnection syndromes include memory and attention [57]. The decrease in these domains has been associated with fatigue and depression [38, 58]. It is possible that emotional deficits seen in mild stroke may also directly represent disconnection syndromes but, to date, have not been demonstrated yet.

## 4. Factors Affecting Mental Health After Mild Stroke

### 4.1. Age At Time Of Stroke

Approximately 12% of strokes occur in adults under 45 years old. Although short term prognosis is considered more favorable due to lower mortality and better functional outcome, recent research suggests that stroke has a more severe impact on psychological and social functioning in this age group [58].

### 4.2. Social Support

Findings across the literature indicate that social support can significantly improve emotional and functional outcomes after stroke [59, 60]. Villain and colleagues (2016) examined the effect of social support immediately following mild stroke and found that higher perceived emotional support was associated with significantly less PSD and more participation in activities of daily living 3 months later [61]. Similarly, material support in the acute phase was associated with performance of activities of daily living (ADLs). Because PSD can have a profound negative impact on rehabilitation and recovery, having sources of both material and emotional support could mean significantly improved outcomes for patients.

Another area of research has examined reciprocal effects of mild stroke between the survivor and the support system. Mild stroke survivors are often directly discharged home and then rely on a family caregiver for support, which can mean sudden and complex changes to the family system with little preparation and a lot of uncertainty. Family caregivers are at higher risk of depression themselves, which can negatively impact their own health as well as the survivor's rehabilitation and recovery (e.g., [62–64]). Importantly, research findings show that emotional well-being after stroke is interdependent in couples, meaning if one partner is depressed, the other is more likely to be depressed as well (e.g., [65]). This highlights the importance of addressing PSD (and overall emotional well-being) in both stroke survivors and their family caregivers.

## 5. Importance of Mental Health Sequelae in Persons with Mild Stroke

While people with mild stroke are able to complete their basic activities of daily living, many report difficulties completing instrumental activities of daily living (IADL). Mental health is a key component affecting IADL performance. As has been shown earlier, mental health issues such as sleep disorders and fatigue affect recovery. In the following sections, we present evidence from the literature to show how unresolved mental health issues in mild stroke lead to decreased participation, inability to return to work, and quality of life.

### 5.1. Participation in Meaningful Activities

Findings from qualitative studies by Carlsson and colleagues [39, 66] suggest that despite appearing to have recovered from their stroke after 1 year, mild stroke survivors reported difficulties in participating in valued activities due to persisting cognitive and emotional dysfunctions. Mental health issues in mild stroke affect participation in meaningful activities and return to work. Using logistic regression analysis, Rozon and Rochette found that the presence of depressive symptoms in the first month after a mild stroke had a statistically significant association with a reduced activity at 6 months after stroke including driving a vehicle and participating in sports/recreational activities [47]. Therefore, it is important to recognize these symptoms and address them as quickly as possible in mild stroke patients.

### 5.2. Return to Work

In a study of 163 mild stroke survivors, depression was found to be significantly higher in those unable to return to work compared to those who returned to work; this study also found that depression was associated with decreased participation [67]. In the same study mentioned above, Rozon and Rochette found that depression symptoms 1 month after a mild stroke were not significantly associated with paid employment at 6 months after stroke [47]. One factor in that finding may be due to the average age of study participants (age 63.3 +/− 12.5 years). Patients from lower socioeconomic backgrounds and in unskilled work are less likely to return to work successfully after a mild stroke [68]. Further research is needed to understand the reasons for this finding.

### 5.3. Quality of Life

Similar to general stroke, PSD has been shown to have a negative impact on functional outcome and quality of life in mild stroke [69]. Research findings suggest that individuals with PSD are less likely to regain their prestroke health status compared with nondepressed counterparts, and having late-onset PSD increases likelihood of poorer physical and mental health [69]. Recovery from depression within 1 year after minor stroke decreases, but does not completely mitigate, the adverse impact of PSD on functional outcome and quality of life.

### 5.4. Secondary Stroke Prevention

Even after a mild stroke, patients are at increased risk to experience a secondary stroke [70]. Secondary stroke prevention requires patients to modify lifestyle factors [71]. Adequate mental health and cognition facilitate the motivation, endurance and planning needed to engage in activities like engaging in exercise, planning healthier meals, and consuming medications as prescribed. Mental health provides a firm foundation from which patients can engage in life long behavior change after mild stroke.

## 6. Strategies to Address Mental Health after Mild Stroke

Healthcare professionals caring for patients with mild stroke are aware of the potential mental health issues, and they should be prepared to educate their patients. There is no assessment designed to assess mental health in mild stroke. Currently, researchers and clinicians use common mental health scales, including Beck's Inventory or PHQ-9, sleep scales such as PQSI, and fatigue scales. These tools seem to be sensitive, but it is unclear whether measurements more specific to mild stroke exist and remain an issue of research interest.

Healthcare professionals may be hesitant to broach the topic of mental health issues with their patients, because of either limited knowledge, time, or even stigma. However, healthcare professionals need to be proactive in identifying mental health issues and should not expect their mild stroke patients to describe them. In the YOU CALL-WE CALL trial, an extremely small number of patients (6.4%) YOU CALL participants availed themselves of the ability to contact a resource person for questions and needs [72].

Knowledge about the influence of psychological factors on quality of life is paramount for clinical practice. Clinicians tend to focus on motor functioning when treating patients with mild stroke; however, as the literature suggests, in spite of mild symptoms and relatively little disability, depression, anxiety, fatigue, and sleep are prominent in mild stroke survivors. Therefore, mental health aspects of recovery and rehabilitation following mild stroke need to be assessed and addressed in order to promote better quality of life.

Clinically, while patients with mild strokes are not excluded from appropriate secondary prevention treatments, they can be overlooked for rehabilitative services as well as hyperacute stroke treatments such as tPA as they are considered “too good to treat.” Yet, as this section suggests, these patients still have major issues which affect their quality of life and overall functioning. Studies such as the PRISMS will help determine the effect of tPA in mild stroke patients and we encourage further acute studies to include this population [73]. In addition, the study design should include standardized mental health assessments to determine where these aspects can be improved with acute stroke treatments.

In the subacute setting, it has been shown that patients can benefit from interventions and rehabilitation. However, traditionally, it is upon the stroke survivor to find these services in the outpatient setting but these patients may need some more guidance. Using a community-based education intervention by nurses, mild stroke survivors demonstrate improved social participation and self-efficacy [74]. In the YOU CALL-WE CALL trial, the active intervention consisting of multimodal support including phone support and education, mild stroke survivors experienced benefit [72].

## 7. Conclusion

Mild stroke and that number will continue to rise with the recent advances in acute stroke treatments. As demonstrated earlier in the paper, mild stroke survivors commonly experience psychosocial impairments such as depression, anxiety, fatigue, and sleep disorders. This is of great interest because of the prevalent belief that persons with mild stroke will make a good recovery and are not getting follow-up services. Furthermore, healthcare professionals do not regularly screen this population for mental health disorders and the screening tools for mental health disorders are not adequately validated in this population. Impairments in mental health will affect mild stroke survivors' ability to do the things they need and want to do such as managing their health, engaging in high risk demanding activities like driving, and returning to work. That is why it is even more important to address mental health in this population.

While there are a lot of gaps in knowledge regarding this topic, our review has uncovered the following points:

- Clinicians need to actively screen their mild stroke patients for these mental health issues. The patients do not seek out help and because they are considered “mild” are not given access to resources.
- There are a few screening tools which can be used.  Table 1 lists some of the common ones.
- Further research is needed to help to determine the best treatments for these deficits.

In summary, mental health disorders are common in mild stroke survivors and adversely affect overall recovery.

## Figures and Tables

**Table 1:** Mental health assessments for use with individuals with mild stroke.

**Depression and Anxiety**	
Patient Health Questionnaire (PHQ-2, PHQ-9, PHQ-15) [7–9]	2-item: only asks about the two hallmark symptoms of depression: depressed mood and anhedonia. Typically used as screen in clinical settings. 9-item: rates the severity of depressive symptoms over the previous 2 weeks by measuring the number of symptoms and their frequency (“not at all” to “nearly every day”). The PHQ-9 is arguably the most popular assessment tool available, having been adopted for a number of different clinical trials, large federally funded surveys, whole federal departments (e.g., Veterans Affairs), and large private groups (e.g., American Heart Association, and American Psychiatric Association). The measure has been validated for use in a large array of medical conditions and is free to use.
Hospital Anxiety and Depression Scale (HADS) [10]	14-item scale that assesses patients for both anxiety and depression. The scale includes two 7-item subscales that were specifically validated for assessing anxiety and depression in individuals with medical comorbidities. The depression subscale focuses on anhedonia, and does not evaluate somatic symptoms. Copyrighted but free for clinical use.
PROMIS-Emotional Distress – Depression [11]	Negative mood (sadness, guilt), views of self (self- criticism, worthlessness), and social cognition (loneliness, interpersonal alienation), as well as decreased positive affect and engagement (loss of interest, meaning, and purpose).
PROMIS-Emotional Distress –Anxiety [12]	Fear (fearfulness, panic), anxious misery (worry, dread), hyperarousal (tension, nervousness, restlessness), and somatic symptoms related to arousal (racing heart, dizziness).
Geriatric Depression Scale [13]	Focuses less on somatic symptoms than on nonsomatic symptoms of depression is the Geriatric Depression Scale (GDS). Free for clinical use. A stroke specific scale has recently been developed [13].
Beck's Depression Inventory II [14]	21-item research tool for measuring symptoms of depression. An abbreviated 7-item version is available for use in a primary care setting. The tool is administered via self-report and requires a paid license for clinical use.
Center for Epidemiological Studies Depression Scale (CESD) [15]	Popular assessment tool that is freely available and has wide applicability in the general population. The scale is based on depressive symptoms used for clinical diagnosis of depression and therefore contains an array of aspects of depression (mood, somatic).
PTSD Checklist Specific for a stressor (PCL-S) [16, 17]	The PCL-S is a validated 17-item scale that corresponds to the DSM-IV criteria for PTSD, with high internal consistency and test-retest reliability. New DSM-5 version has been developed [18].

**Fatigue**	
PROMIS-Fatigue [11, 19]	Range of symptoms, from mild subjective feelings of tiredness to an overwhelming, debilitating, and sustained sense of exhaustion that likely decreases one's ability to execute daily activities and function normally in family or social roles.
Fatigue Severity Scale [20]	A self-report questionnaire that assesses the severity of fatigue and its impact on people's functioning in everyday life. The FSS has been validated for use in post-stroke fatigue [21].

**Sleep**	
Pittsburgh Seep Quality Index (PSQI) [22]	24-item scale that measures sleep disturbances along 7 dimensions: subjective sleep quality, sleep latency, sleep duration, habitual sleep efficiency, sleep disturbances, use of sleep medication, and daytime dysfunction.
Global sleep assessment questionnaire (GSAQ) [23]	11 items asking about symptom frequency over the last four weeks covering mood, life activities and medical issues as they relate to sleep, along with symptoms associated with insomnia, obstructive sleep apnea, restless legs syndrome/periodic limb movement, and parasomnias. Comprehensive screening tool [24].
PROMIS-Sleep Disturbance [11, 19]	Perceptions of sleep quality, sleep depth, and restoration associated with sleep.
PROMIS-Sleep-Related Impairment [11]	Perceptions of alertness, sleepiness, and tiredness during usual waking hours, and the perceived functional impairments during wakefulness associated with sleep problems or impaired alertness.

## Abbreviations

PSD: Poststroke depression
PTSD: Posttraumatic stress disorder
TIA: Transient ischemic attacks
ADLs: Activities of daily living.
